# Plant Proteoforms Under Environmental Stress: Functional Proteins Arising From a Single Gene

**DOI:** 10.3389/fpls.2021.793113

**Published:** 2021-12-14

**Authors:** Klára Kosová, Pavel Vítámvás, Ilja Tom Prášil, Miroslav Klíma, Jenny Renaut

**Affiliations:** ^1^Division of Crop Genetics and Plant Breeding, Crop Research Institute, Prague, Czechia; ^2^Biotechnologies and Environmental Analytics Platform (BEAP), Environmental Research and Innovation (ERIN) Department, Luxembourg Institute of Science and Technology (LIST), Esch-Sur-Alzette, Luxembourg

**Keywords:** protein isoforms, protein posttranslational modifications (PTMs), protein-protein interactions, biological functions, environmental stresses, crops

## Abstract

Proteins are directly involved in plant phenotypic response to ever changing environmental conditions. The ability to produce multiple mature functional proteins, i.e., proteoforms, from a single gene sequence represents an efficient tool ensuring the diversification of protein biological functions underlying the diversity of plant phenotypic responses to environmental stresses. Basically, two major kinds of proteoforms can be distinguished: protein isoforms, i.e., alterations at protein sequence level arising from posttranscriptional modifications of a single pre-mRNA by alternative splicing or editing, and protein posttranslational modifications (PTMs), i.e., enzymatically catalyzed or spontaneous modifications of certain amino acid residues resulting in altered biological functions (or loss of biological functions, such as in non-functional proteins that raised as a product of spontaneous protein modification by reactive molecular species, RMS). Modulation of protein final sequences resulting in different protein isoforms as well as modulation of chemical properties of key amino acid residues by different PTMs (such as phosphorylation, *N*- and *O*-glycosylation, methylation, acylation, *S*-glutathionylation, ubiquitinylation, sumoylation, and modifications by RMS), thus, represents an efficient means to ensure the flexible modulation of protein biological functions in response to ever changing environmental conditions. The aim of this review is to provide a basic overview of the structural and functional diversity of proteoforms derived from a single gene in the context of plant evolutional adaptations underlying plant responses to the variability of environmental stresses, i.e., adverse cues mobilizing plant adaptive mechanisms to diminish their harmful effects.

## Introduction

**FIGURE 1:**
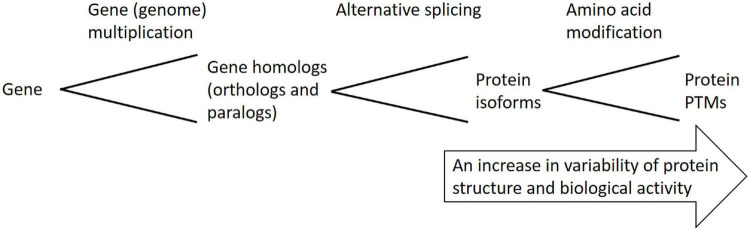
Schematic overview of posttranscriptional and posttranslational mechanisms leading to multiple functional proteoforms arising from a single gene.

Protein versatility reflects the versatility of plant phenotypes, since proteins are directly involved in biological processes shaping plant phenotypes. Plant genome sequencing programs revealed a relatively low number of protein-coding genes (e.g., around 27,000 protein-coding genes in *Arabidopsis thaliana* genome) with respect to the number of different functional proteins present in plant cells, tissues, or even at whole-organism level [more than 100,000 different functional protein species and around 25 billion protein molecules are estimated to be present in Arabidopsis mesophyll cell by Heinemann et al. (2020)], thus, it is evident that complexity at the levels “gene” → “transcript” → “protein” rises due to the fact that a single gene can produce multiple functional proteins *via* an array of posttranscriptional and posttranslational modifications (Figure 1). It is, thus, evident that plant response to environmental cues at proteome level cannot be simply derived from transcriptome data. All proteins encoded by a single gene are termed proteoforms (Smith et al., 2013).

Basically, two major categories of functional proteins derived from a given gene can be distinguished: “protein isoforms,” which differ in their primary protein sequence, and “protein posttranslational modifications (PTMs),” which differ in modifications of side amino acid residues ranging from a few atoms (nitrosylation, carbonylation) to whole peptides (*S*-glutathionylation, ubiquitinylation, sumoylation). In addition, alterations in protein/enzyme conformation in response to ligand/substrate interaction can be considered another kind of proteoforms. Differences in primary protein sequences among protein isoforms lead to differential molecular weight (MW), while conjugation of charged molecules or residues leads to altered isoelectric point (pI) values in differential PTMs. Since differential protein isoforms and PTMs often acquire differential biological functions, studying different protein isoforms and PTMs is of high importance for understanding protein functions. Methods based on 2-DE and its modifications are suitable for studies on protein isoforms and PTMs because of protein separation according to pI and MW values. Moreover, some specialized 2-DE gel-based protocols on PTMs detection were developed including either specific staining such as phosphoprotein detection by ProQ Diamond stain or PTM-specific primary antibody such as immunoblot detection of carbonylated proteins modified by dinitrophenylhydrazine (DNPH) to dinitrophenylhydrazones by a specific primary antibody [Oxyblot kit; Levine et al. (1990)], biotin-switch technique (BST) for detection of *S*-nitrosylated proteins (Jaffrey and Snyder, 2001) or concanavalin A lectin blot for glycoprotein detection (Hashiguchi and Komatsu, 2016) and immunoprecipitation methods for S-glutathionylated proteins detection (Butturini et al., 2019). However, the dominant methods for proteoforms detection represent MS/MS-based approaches, such as TMT labeling for phosphoprotein detection. Recently, an excellent review on MS-based approaches used for PTM identifications was published by Virág et al. (2020). Since only a relatively small fraction of a given protein undergoes PTM, the modified proteins reveal low abundances and have to be enriched in the protein samples; for example, ZrO_2_- or TiO_2_-IMAC beads are used for phosphoprotein enrichment. Approaches of immune affinity chromatography using specific anti-PTM primary antibodies are also widely used for PTM enrichment. However, the differential accumulation patterns and biological functions of different proteoforms that raised from a single gene indicate an urgent need to study plant responses to environmental cues at the proteome and PTMome levels to understand the plant phenotype under stress.

Plants as sessile organisms have developed evolutionary mechanisms to adapt to adverse environmental conditions imposing stress responses. Stress-adaptive responses involve several alterations in plant water regime (dehydration stress), redox homeostasis (enhanced risk of redox stress as a consequence of alterations in plant metabolism leading to enhanced levels of RMS) and altered physio-biochemical properties of biomolecules (e.g., altered fluidity of cellular membranes under low or high temperatures). Proteomics studies found out that these alterations are underlied by differences at proteome level including not only differential protein relative accumulation, but also differential spectrum of proteoforms.

Recently, it became evident that stress-induced alterations in protein relative abundance represent just one aspect of plant proteome stress responses, and that modifications at posttranscriptional and posttranslational levels leading to differential protein isoforms and PTMs, respectively, add another layer to the complexity and fine tuning of the plant stress response (Barua et al., 2019). The impacts of differential protein abundance patterns, structural modifications, such as isoforms and PTMs, cellular localization, and interacting partners in plant responses to environmental stresses were already discussed in recent reviews (Wang and Komatsu, 2016a; Kosová et al., 2018, 2021). Basic literature information on most studied protein PTMs in crop plants under abiotic stress was reviewed by Hashiguchi and Komatsu (2016) and Mustafa and Komatsu (2021) with a major focus on protein phosphorylation, glycosylation, acetylation, and succinylation, and by Wu et al. (2016) with a major focus on phosphorylation, ubiquitination and sumoylation, and most studied redox modifications such as protein carbonylation and *S*-nitrosylation. Recently, the role of PTMs in plant-pathogen interactions was reviewed by Gough and Sadanandom (2021) with a focus on phosphorylation, ubiquitination, and SUMoylation. Specialized reviews on plant protein phosphorylation under both abiotic and biotic stress signaling (Rampitsch and Bykova, 2012), rice phosphoproteomics studies (Ajadi et al., 2020), sumoylation (Benlloch and Lois, 2018), and nitrosylation (Romero-Puertas et al., 2013; Feng et al., 2019) were also published. A review focused on selected posttranscriptional and posttranslational regulations of drought and heat stress responses in plants with a focus on alternative splicing, RNA-mediated silencing, and ubiquitination and sumoylation protein modifications was published by Guerra et al. (2015).

The aim of this review is to provide a basic overview of the diversity and versatility of proteoforms derived from a single gene in order to emphasize the necessity of studying differences in protein isoforms and PTMs to understand protein biological functions. Proteoforms, i.e., multiple functional proteins derived from a single gene, highlight the necessity to study plant phenotypic response at proteome level, since it cannot be simply derived from transcriptome data. Examples of the impacts of diverse posttranscriptional and posttranslational modifications on the diversity of proteoforms arised from a single gene with respect to their biological functions are provided.

## Protein Isoforms

Protein isoforms can either arise from a single gene *via* posttranscriptional modifications such as alternative splicing or RNA editing or they can represent products of homologous genes including both orthologous and paralogous genes. Orthologous genes represent homologs of a given gene in genomes of another plant species, while paralogous genes represent homologous genes that are products of the local multiplication of a given sequence in a single genome. As an example of gene paralogs, cluster of *CBF* genes at *Fr2* locus on the long arm of homoeologous group 5 chromosomes in Triticeae can be given (Tondelli et al., 2011). Several important crops, such as bread wheat, potato, oilseed rape, banana, and others, which are allopolyploids, contain whole sets of homeologous genomes; thus, they contain homeologous genes. Homeologs are pairs of genes that originated by speciation and are brought back together by allopolyploidization (Glover et al., 2016). In addition to sequence homeology, the array of protein isoforms can be significantly increased by the mechanism of alternative splicing, giving rise to multiple functional proteins from a single gene. Alternative splicing, thus, represents an efficient evolutionary mechanism of enhancing phenotypic variation in response to environmental cues (Shang et al., 2017). Mechanisms of isoform differentiation at the transcript level are not included in this study, since we focus on proteins and their biological functions.

Protein isoforms can reveal differential biological functions because of differential expression patterns, differential cellular localization, differential protein interaction partners, and others. Some examples of functional diversification of isoformic proteins are provided below:

Transcriptome and proteome reprogramming under stress: non-canonical histone isoforms were found to play important roles in transcriptome reprogramming under stress. Alterations in transcript expression between canonical H2A and cold-induced H2AZ histone isoforms affecting nucleosome structure and DNA wrapping underlying transcriptome remodeling under cold acclimation were observed in *A. thaliana* (Kumar and Wigge, 2010) as well as in winter barley crowns (Janská et al., 2011). At proteome level, Martinez-Seidel et al. (2021) reported differential ribosomal proteins paralogs underlying alterations at ribosomal level and proteome reprogramming in barley root tip meristems subjected to cold acclimation.

Differential gene copy number determining quantitative differences in gene expression and quantitative phenotypic traits: Knox et al. (2010) and Würschum et al. (2017) revealed that the quantitative differences in acquired frost tolerance achieved by winter vs. spring Triticeae genotypes are associated with a higher copy number of *CBF* genes encoded by frost-tolerant winter genotypes with respect to the spring ones.

Differential expression patterns and differential interaction protein partners: Hsc70 protein is known as a cochaperone involved in protein folding. Hsc70-1 and Hsc70-3 isoforms are constitutively expressed, while Hsc70-2 isoform is pathogen-inducible and forms a complex with SGT1 protein involved in R-gene mediated resistance (Noël et al., 2007).

RubisCO activase (RCA) is crucial for maintaining the high carboxylation activity of RubisCO, since it removes pentosephosphates inhibiting the formation of carbamate critical for RubisCO carboxylation activity from RubisCO active site. Wang et al. (2020) studied RCA isoforms in relatively heat-tolerant *Rhododendron hainanense*. It encodes 3 RCA homologous genes named RCA1, RCA2, and RCA3, and each of them can give rise to multiple isoforms *via* a mechanism of alternative splicing (RCA1, RCA2 and RCA3 can potentially encode 5, 3, and 2 isoforms, respectively). While RCA2 and RCA3 isoforms are constitutively expressed, RCA1 isoforms are heat-inducible (especially the RCA1-X1 isoform) and persist in plants in recovery following a heat stress treatment; thus, they seem to provide some type of epigenetic stress memory involved in heat stress acclimation; enhanced levels of RCA1 persisting in plants reveal an improvement effect on plant photosynthesis when exposed to stress (Wang et al., 2020).

Differential cellular localization: Differential functions were described for nuclear isoforms and cytoplasmic (plastid, mitochondrial) isoforms of glycolytic enzymes. The nuclear isoform of fructose-bisphosphate aldolase (FBA) is known to act as a DNA-binding protein involved in regulation of expression of its own gene as well as other genes (Ronai et al., 1992). The nuclear isoform of glyceraldehyde-3-phosphate dehydrogenase (GAPDH) is known to act as a tRNA-binding protein involved in tRNA export (Singh and Green, 1993). It has also been reported that FBA may be involved in the integration of intra- and extracellular signals associated with growth, development, and sugar biosynthesis (Li et al., 2012), and that non-the phosphorylating isoform of GAPDH may be involved in regulation of ROS levels (Bustos et al., 2008). The nuclear isoform of enolase encoded by *Los2* locus in *A. thaliana* functions as a transcriptional repressor of STZ/ZAT10, which is a repressor of cold-inducible CBF pathway; the nuclear isoform of enolase, thus, acts as an indirect positive regulator of CBF pathway and CBF-regulated *COR* gene expression (Lee et al., 2002). Tonoplast-associated isoforms of aldolase and enolase are involved in the activation of V-ATPase in salt-treated *Mesembryanthemum crystallinum* plants, leading to Na^+^ vacuolar accumulation (Barkla et al., 2009).

Differential regulatory functions: Cytoplasmic eIF5A is known as a translation initiation factor involved in the regulation of protein biosynthesis. However, nuclear eIF5A isoforms named eIF5A-1 and eIF5A-2 were reported to be involved in the regulation of cell cycle with eIF5A-1 involved in a “switch” between cell proliferation and cell death, with eIF5A-1 leading to programmed cell death (PCD) while eIF5A-2 enhancing cell proliferation (Thompson et al., 2004).

Different cofactors and cellular localizations: Upon stress, a risk of ROS production in aerobic metabolism increases because of imbalances between the kinetics of electrontransport processes (respiratory electron transport chain, photosynthetic electrontransport chain) and the following enzymatic reactions. To diminish the harmful effects of ROS overproduction, plants express a wide array of several redox metabolism-related enzymes in order to finely tune their final levels. ROS levels are, thus, controlled by different peroxiredoxin (Prx) and thioredoxin (Trx) isoforms ensuring a fine tuning of redox processes in photosynthesis and respiratory electron transport chains in inner chloroplast and mitochondrial membranes, respectively. Superoxide dismutase (SOD) isoforms represent a well-known ROS scavenging enzyme with different cofactor ions and cellular localizations: Cu/Zn-SOD-cytoplasmic; Fe-SOD-plastid; Mn-SOD-mitochondrial localization. Several isoforms of ROS-scavenging enzymes, thus, provide fine tuning and control of oxidative stress, which is ubiquitously associated with aerobic metabolism localized to semiautonomous organelles, i.e., photosynthetic and respiratory electron transport chains localized in inner chloroplast and mitochondrial membranes, respectively (Meyer et al., 2005; Dietz, 2016).

Different isoforms form different cellular structures and reveal differential protein-protein interactions: Kijima et al. (2018) studied differential actin isoforms in *A. thaliana* and found that the *A. thaliana* genome encodes eight actin isoforms, out of which three (AtACT2, AtACT7, and AtACT8) are expressed at the vegetative stage while the remaining five (AtACT1, AtACT3, AtACT4, AtACT11, and AtACT12) are expressed at reproductive stage. They also found out that two vegetative actin isoforms, AtACT2 and AtACT7, form different types of filaments, with AtACT2 forming thinner filaments while AtACT7 forming thick bundles. They also reveal a different expression upon stress and interact with different actin-binding proteins (ABPs).

Examples of functional diversification of plant protein isoforms in plant stress responses are given in Table 1.

**TABLE 1:** Examples of cellular and functional diversification of protein isoforms in response to environmental stresses.

Factors determining protein isoforms	References
Transcriptome reprogramming under cold: histone H2AZ instead of canonical H2A isoform → altered pattern of euchromatin vs. heterochromatin	Kumar and Wigge, 2010
Cellular localization—different cofactor:	
SOD isoforms: Cu/Zn-SOD—cytoplasmic; Mn-SOD—mitochondrial;	Kosová et al., 2018
Fe-SOD—chloroplast	
Cellular localization—different function: cytoplasmic (C) vs. nuclear (N) protein isoforms	
eIF3: translation regulation (C) × cell cycle regulation (N)	Thompson et al., 2004
enolase: glycolytic enzyme (C) × transcriptional regulator (N	Lee et al., 2002
Fine tuning of biological processes—isoforms with overlapping functions:	
Peroxiredoxin and thioredoxin isoforms—better regulation of redox stress in photosynthetic electron transport chain (ETC)	
Enhanced *CBF* gene copy number in winter vs. spring barley genotype → enhanced low-temperature (LT) tolerance in winter-type plants	Knox et al., 2010
Different expression patterns and interacting partners: Hsc70-1, Hsc70-3—constitutive; Hsc70-2—pathogen-inducible, interacting with SGT1 involved in R-gene mediated resistance	Noël et al., 2007

## Protein Posttranslational Modifications

Protein posttranslational modifications (PTMs) involve all modifications of side amino acid residues occurring in nascent proteins and ranging from a few atoms, such as carbonyl or nitrosyl groups to whole peptides such as *S*-glutathione, ubiquitin, and SUMO. Currently, over 300 different PTMs were reported, such as phosphorylation, *N*- and *O*-glycosylation; reactive molecular species (RMS)-related modifications, such as glycation, carbonylation, nitrosylation, and others; peptide modifications, such as ubiquitylation and sumoylation, as well as acylation modifications of lysine residues such as succinylation (Xu W. et al., 2017) and crotonylation (Xu Y. et al., 2017). Protein PTMs range from single-step spontaneous non-enzymatic amino acid modifications by RMS, such as protein carbonylation or nitrosylation to large PTMs resulting from highly coordinated multi-step enzymatic processes, such as protein *N*-glycosylation or ubiquitination. An overview of most studied PTMs, such as target amino acids and added functional groups, is given in Table 2. Since recent articles revealed differential alterations in plant total proteome and PTMome in response to a given biological process (He et al., 2020), it is necessary to study plant responses to a given cue such as environmental stresses at PTMome level. Different PTMs result in differential modulation of protein biological functions; for example, regarding E3 RING-type ubiquitin ligase PARKIN, it was reported that nitrosylation decreases while persulfidation increases its ubiquitination capacity in mammalian neurons (Vandiver et al., 2013).

**TABLE 2:** Types of protein posttranslational modifications (PTMs), such as reactive agents, target amino acids, and resulting adducts, and methods used for PTM detection.

PTM type	Agent	Target amino acid	Detection method (references)
Phosphorylation	ATP (γ-phosphate group)	Ser, Thr, Tyr; His	ProQ Diamond gel staining ZrO_2_/TiO_2_ based enrichment, TMT labeling
Glycosylation	NDP-monosaccharide (e.g., GDP-mannose, UDP-glucose)	*N*-glycosylation: Asn in Asn-X-Ser/Thr motif (X represents any amino acid except Pro) *O*-glycosylation: Ser, Thr	Concanavalin A lectin blot
Acylation Short-chain acids: acetylation, propionylation, succinylation, malonylation, crotonylation) Long-chain acids: palmitoylation, myristoylation	Activated acyl (acyl-CoA)	Lys (ε-amino group)	Anti-acyl-lysine antibody (immunoblots, immune affinity enrichment of acylated proteins prior to LC-MS/MS)
Methylation	*S*-adenosylmethionine (SAM)	Arg; Lys (ε-amino group)	ADMA- and SDMA-, anti-methyl antibodies (immune affinity enrichment LC-MS/MS) (Carlson and Gozani, 2014)
Peptide conjugation			
*S*-glutathionylation	*S*-glutathione	Cys (thiol group)	Anti-*S*-glutathione antibody precipitation; biotin-GSH labeling
Ubiquitination	Ubiquitin (diglycine motif at C-terminus)	Lys (ε-amino group)	Anti-ubiquitin specific antibody (immunoblot)
Sumoylation	SUMO (diglycine motif at C-terminus)	Lys (ε-amino group)	Immunoprecipitation with anti-SUMO-1/2/3 antibodies (Sarge and Park-Sarge, 2009)
RMS related PTMs			
Glycation	Active mono- (acrolein) and dicarbonyls (glyoxal, methylglyoxal, reducing sugars)	Lys, Arg (amino group)	Oxyblot kit (carbonyl derivatization to difenylhydrazones and their detection by specific antibody)
*S*-nitrosylation *O*-nitrosylation	NO (GSNO), ONOO-, NO_2_	Cys Tyr	Biotin-switch (immunoblot) (Jaffrey and Snyder, 2001)
Oxidation (ROS)	O_2_-, H_2_O_2_, OH, 1O_2_	Arg, Lys, Pro, Thr	
Sulfurylation (RSS; persulfidation)	H_2_S	Cys (thiol group)	Tag-switch LC-MS/MS

*ADMA, asymmetric dimethylated arginine; SDMA, symmetric dimethylated arginine.*

When studying protein PTMs, it has to be kept in mind that only a fraction of a given protein undergoes a given PTM and that the given PTM could be reversible and transient, i.e., to occur for a limited time, as it is evident for proteins in signaling cascades associated with transient phosphorylation/dephosphorylation events or for some RMS-mediated PTMs, which act as a signal indicating plant protein damage under stress. Moreover, a given amino acid residue can undergo multiple PTMs depending on the kind of stress; thus, the problem of PTM crosstalk has to be studied. In addition, only a few studies dealt with the impacts of combined stress treatments, which are common in the field conditions on plant protein PTM patterns. The results of the few studies indicate that plant response to combined stress is associated with a unique PTM pattern in comparison to the effects of single stress factors (Hu et al., 2015b).

The concept of PTM “writers,” “erasers,” and “readers” was proposed by Leutert et al. (2021). PTM mechanisms consist of “writers,” i.e., enzymes catalyzing the addition of the PTM group to a target amino acid residue (kinases, ubiquitin ligases, acylases,…); “erasers,” i.e., enzymes catalyzing reversible removal of a given PTM from a target amino acid residue (phosphatases, deubiquitinases, deacetylases,…); and “readers,” i.e., all proteins interacting with the given PTM.

Accumulation of the data from tandem MS/MS identifications on protein PTMs led to the development of specific databases and prediction tools for most studied PTMs. Plant protein PTMs are specifically mapped by the Plant PTM Viewer^1^, an integrative PTM resource database providing data on 19 PTM types (Willems et al., 2019), which currently contains PTMs data from five plant species, *A. thaliana* (165,279 PTMs in 55,988 proteins), *Chlamydomonas reinhardtii* (18,070 PTMs in 5,951 proteins), *Oryza sativa* ssp. *japonica* (56,606 PTMs in 19,500 proteins), *Triticum aestivum* (53,580 PTMs in 25,150 proteins), and *Zea mays* (143,869 PTMs in 37,099 proteins) (accessed 3rd November, 2021). MultiPTMs prediction tools, such as PTMscape (Li et al., 2018) and PTM-ssMP web server (Liu et al., 2018) utilizing information on site-specific profiles and enabling the identification of potential target motifs for multiple kinds of PTMs such as phosphorylation, acetylation, methylation, *O*-glycosylation, ubiquitination, and sumoylation, are available. Moreover, prediction tools for the most studied PTMs, such as PhosphoSitePlus (PSP) (Hornbeck et al., 2015), PHOSIDA (Gnad et al., 2011) for phosphoprotein detection, and UbiSite, as a web tool for ubiquitination prediction (Huang et al., 2016), etc., are available.

A brief overview of major high-throughput proteomic studies focused on stress-responsive PTMomes is provided in Table 3. In addition to the PTMs described below, other PTMs, such as protein C-terminal amidation or monomeric and polymeric ADP-ribosylation (MARylation and PARylation, linear or branched), are emerging to play an important role in plant innate immunity and plant-bacterial pathogen interactions. However, elucidation of their roles in plant responses to bacterial pathogens and biotic stress deserves further study (Feng et al., 2016).

**TABLE 3:** Overview of high-throughput proteomic studies focused on PTMome analysis in stress-treated plants.

Plant species	Stress treatment	Method	Differentially abundant proteins with PTM	References
Phosphorylation				
*Arabidopsis thaliana* Col—4 week old plants	Salinity (200 mM NaCl, 3 day)	Zr^4+^-IMAC beads PP enrichment; LC-MS/MS	15 phosphoproteins in membrane fraction: aquaporins PIP2;1, PIP2;4; 14-3-3 interacting protein AHA-I	Hsu et al., 2009
*Arabidopsis thaliana—*12 day old seedlings	800 mM mannitol, 100 μM ABA (30 min.)	Phosphopeptide enrichment with PolyMAC-Ti, nanoLC HPLC, LTQ-Orbitrap	1,850 quantified phosphopeptides, 468 differentially regulated: MAPK, CKII, SnRK2.1, SnRK2.4, SnRK2.5, SnRK2.10	Xue et al., 2013
Chickpea (*Cicer arietinum*) cv. JG-62	Drought	2-DE Pro-Q Diamond staining; immunoblots (anti-phosphoserine, antiphospho-threonine, antiphospho-tyrosine antibodies	Regulatory and functional proteins: photosynthesis (OEE1, CA, ATP synthase subunit α) and photorespiration, stress and defense, molecular chaperones, ion transport—CaDREPP1—plasma membrane polypeptide family protein	Subba et al., 2013
		TMT labeling and TiO_2_ phosphopeptide enrichment	Kinases, phosphatases, spliceosome components	Barua et al., 2019
Common bean VAX1 root tip	Osmotic stress (PEG-6000)	2DE ProQ Diamond staining	10 PPs: Ser/Thr-specific PP2A; PRK; DHN; actin; lactoylglutathione lyase;	Yang et al., 2013
Soybean cv. Enrei root tip—nuclei—2 day old seedlings	Flooding: 3 h	Polymer-based metal ion affinity phosphopeptide enrichment; nanoLC-MS/MS	ABA-responsive proteins: zinc finger/BTB domain containing protein 47, GRP, rRNA processing protein Rrp5	Yin and Komatsu, 2015
*Brachypodium distachyon* Bd21 at 3-leaf stage	Osmotic stress (20% PEG-6000 in Hoagland solution) 48 h	TiO_2_ IMAC beads enrichment; LC-MS/MS	Signaling (MAPK cascade, SnRK2, CDPK, PP2C), TFs (NF-Y, Hsf), ion transport (PIP, NIP, H^+^-ATPase in plasma membrane), stress (DHN3-like, HSP90)	Yuan et al., 2016
Barley cv. Haruna-nijyo	Salinity (200 mM NaCl) 24 h	qRT-PCR	10 HvPIP aquaporins (HvPIP2)	Horie et al., 2011
Einkorn wheat (*Triticum monococcum*) 3-leaf stage	Salinity (0, 80, 320 mM NaCl)	2-DE and Pro-Q Diamond phosphoprotein staining MALDI-TOF/TOF	20 phosphoproteins identified: cp31BHv, betaine-aldehyde dehydrogenase, leucine aminopeptidase 2, redox enzymes (Cu/Zn-SOD, 2-Cys Prx BAS1), chaperone (CPN60α), photosynthesis (OEE), ATP synthesis (ATP synthase CF1β)	Lv et al., 2016
Wheat (*Triticum aestivum*) Zhongmai 175	Drought	Pro-Q Diamond phosphoprotein staining, 2-DE, MALDI-TOF/TOF	Wheat flag leaf: photosynthesis-related (LHCII), dephosphorylation of RCA under drought; Phosphorylation of glycolysis enzymes (FBA, GAPDH, ENO), TCA cycle	Luo et al., 2018
Zhengmai 366	Salinity (180 mM NaCl) 24 h	2-DE and Pro-Q Diamond phosphoprotein staining	Wheat embryo and endosperm: 14 PPs in embryo, 6 PPs in endosperm: COR, 27K protein, SOD	Luo et al., 2019
Hanxuan 10, Ningchun 47—3-leaf stage	Osmotic stress (20% PEG-6000)	TiO_2_ beads microcolumn enrichment, LC-MS/MS	Wheat seedling leaves: 31 PPs involved in RNA transcription/processing, stress and defense, signal transduction	Zhang et al., 2014a
Henong 341—flag leaf and grains at 28 day after flowering	Drought (decreased SWC)	TiO_2_ beads microcolumn enrichment, LC-MS/MS	Wheat grain: 63 PPs—signaling (LRR-receptor kinases, casein kinase I, CDK, CDK-like); chaperones (HSPs), starch biosynthesis	Zhang et al., 2014b
Yangmai 18—spike	*Fusarium graminearum*	2-DE, immunoblots (anti-phosphoserine, anti-phosphothreonine, anti-phosphotyrosine antibodies), MALDI-TOF MS	28 PPs: signaling (receptor protein kinase PERK1-like); RING-finger E3 ubiquitin ligase; dnaJ-like protein; ADP ribosylation factor; metabolism (PGK; cinnamoyl-CoA reductase, isochorismate synthase); transport (ABC transporter); stress/defense (cytochrome P450)	Ding et al., 2016
Rice (cv. Nipponbare)	Salinity	2-DE ProQ Diamond	Up: Putative ribosomal protein S29, HSP70, MRL Down: ATP synthase β, glucan endo-1,3-β-glucosidase	Chitteti and Peng, 2007
	Drought		10 PPs: NAD-MDH, ribosomal protein, r40c1 protein, *S*-like ribonuclease, ethylene-inducible protein, GLP1, ABA-inducible protein	Ke et al., 2009
Rice cv. Zhong Jiazao-17 indica rice—12 day seedlings	10, 100 μM CdCl_2_*2.5 H_2_O	TiO_2_-IMAC enrichment, LC-MS/MS	1,244 PPs, 392↑—Cd: ABA signaling (PP2C30, PP2C66), CDPK signaling, MAPK signaling; WRKY TFs, 14-3-3 like, NADH dehydrogenase, LHCB	Zhong et al., 2017
D69 (S), D28 (T)—3-leaf stage	0.1 mmol dm^–3^ Cd	2-DE MALDI-TOF/TOF	Carbon metabolism, proteolytic enzymes, F-box containing TFs, zinc-finger, MYB TFs, DEAD-box ATP-dependent RNA helicase 37	Fang et al., 2019
Maize cv. Zhengdan 958—5-leaf stage	Drought (PEG -−0.7 MPa, 8 h), heat (42°C, 1 h) and combined stress	TiO_2_-IMAC beads PP enrichment; iTRAQ, nano LC-MS/MS	Stress proteins: HSPs; Receptor proteins: Ca-dependent receptor, gibberellin receptor GID1	Hu et al., 2015b
Maize mutant vp5 (ABA deficient)—2 week old	Osmotic stress (−0.7 MPa PEG6000, 8 h)	TiO_2_-IMAC beads PP enrichment; iTRAQ, nanoLC-MS/MS	4,052 phosphopeptides—3,017 phosphoproteins; 14 categories: signaling (receptor-like protein kinase; tpa: protein kinase superfamily protein, leucine-rich repeat protein kinase, CDPK); chloroplast PPs (CPN60β; stresschloroplastic like X2 isoform); E3 ubiquitin protein ligase	Hu et al., 2015a
Maize inbred line B73—5-leaf stage	Drought (mild and severe water deficits) and recovery (5–60 min)	TiO_2_-IMAC beads PP enrichment; nano LC-MS/MS	Identified phosphoproteins: cell division and expansion related (GAP, USP, Golgi SNARE12), carbohydrate metabolism (SuSy3), chromatin remodeling (histone deacetylase 2b, HDT2), ABC-transporter, DEAD-box ATP-dependent RNA helicase 52C, BZIP, RING finger protein 126	Bonhomme et al., 2012
Sugarbeet monosomic addition line M14—3 week	Salinity (0, 200, 400 mM NaCl, 10, 30, 60, 90 min.)	Nutip PP enrichment; LC-MS/MS	Signaling: CDPK, MAPK, 14-3-3; calcium binding protein CML35; Ras-related smallGTP binding protein	Yu et al., 2016
Okra (*Abelmoschus esculentus*) cv. Wufu leaves	Salinity (300 mM NaCl, 48 h)	Affinity enrichment, TMT kit, LC-MS/MS	Photosynthesis antenna proteins (LHCII), RNA degradation	Yu et al., 2019
*N*-glycosylation				
Soybean (cv. Enrei) root tip—2 day old	Flooding (2 day)	SDS-PAGE and concanavalin A lectin blot	Up: glycolysis enzymes (GAPDH) Down: *N*-glycan synthesis enzymes (STT3)	Mustafa and Komatsu, 2014
Rice (cv. Nipponbare) leaf sheath—2 week old seedlings	Cold (5°C, 2 day)	SDS-PAGE and concanavalin A lectin blot	Up: energy metabolism (mitochondrial F1-ATPase, 6-phosphogluconate dehydrogenase, NADP-dependent malic enzyme, enolase, UDP-glucose pyrophosphorylase), chaperone (CPN60α, HSP90), signaling (calreticulin)	Komatsu et al., 2009
Carbonylation				
Rice cv. Malviya-36 (S), Vandana (T)	15% PEG-6000, 1 mM AlCl_3_	2DE, immunoblot of DNPH-derivatized proteins	Up: RubisCO LSU, OEE3, PS I subunit II, IV; CaM	Pandey and Dubey, 2021
*S*-nitrosylation				
*Brassica juncea* var. Pusa Jaikisan 7 day old seedlings	Cold (4°C) for 2–96 h	2-DE, biotin-switch	20 *S*-nitrosylated proteins: RubisCO LSU, SSU; ACC synthase 4, PRX, FBA, HSP70, APX, SOD	Abat and Deswal, 2009
		RubisCO depletion, 2DE, biotin switch assay, nLC-MS/MS	*S*-nitrosylated proteins: SOD, Trx-H type; GAPDH, FBA, sedoheptulose-bisphosphatase	Sehrawat et al., 2013
		RubisCO depletion, 2DE, biotin switch assay, MALDI-TOF/TOF, nLC-MS/MS	48 (24 GSNO-treated, 24 cold-responsive) *S*-nitrosoproteins: 12S globulin protein cruciferin; redox (DHAR, Cu/Zn-SOD), stress defense (lactoylglutathione lyase, GLP)	Sehrawat and Deswal, 2014
Citrus (*Citrus aurantium*) mature leaves from 5-month old plants	150 mM NaCl, 16 day	2-DE, immunoblots (carbonylation, Tyr-nitration), biotin-switch (*S*-nitrosylation)	Leaves: Carbonylation: RCA, Tyr-nitration: RubisCO LSU, *S*-nitrosylation: PRX	Tanou et al., 2009
	150 mM NaCl, 8 day	1-DE, immunoblots (carbonylation, Tyr-nitration), biotin-switch (*S*-nitrosylation)	Leaves: Fe-SOD, NOX, AOX, GSNOR, Roots: Cu/Zn-SOD, Mn-SOD, NOX, DAO, PAO, GSNOR	Tanou et al., 2012
	Continuous light, dark, heat, cold, drought, salinity	SDS-PAGE and immunoblots (biotin switch assay)	*S*-nitrosylation patterns enhanced by heat, cold and drought but suppressed by dark and salinity with respect to control	Ziogas et al., 2013
Pea (*Pisum sativum*) cv. Lincoln—leaf mitochondria	150 mM NaCl, 14 day	SDS-PAGE, MALDI-TOF/TOF, immunoblot	ATP synthase β, HSP90, PRXIIF	Camejo et al., 2013

*AOX, alternative oxidase; APX, ascorbate peroxidase; CA, carbonic anhydrase; CaM, calmodulin; CDK, cyclin-dependent kinase; CDPK, calcium-dependent protein kinase; CK, casein kinase; DHAR, dehydroascorbate reductase; DHN, dehydrin; ENO, enolase; FBA, fructose bisphosphate aldolase; GAP, GTPase-activating protein; GAPDH, glyceraldehyde-3-phosphate dehydrogenase; GLP, germin-like protein; GRP, glycine-rich protein; GSNO, S-nitrosoglutathione; GSNOR, S-nitrosoglutathione reductase; LHC, light-harvesting complex; MDH, malate dehydrogenase; OEE, oxygen-evolving enhancer protein; PGK, phosphoglycerate kinase; PP, protein phosphatase; PS, photosystem; PRK, pyruvate kinase; PRX, peroxiredoxin; RCA, RubisCO activase; RubisCO (LSU, SSU), ribulose bisphosphate carboxylase/oxygenase (large and small subunit); S, susceptible genotype (to a given stress); SOD, superoxide dismutase; SuSy, sucrose synthase; SWC, soil water content; T, tolerant genotype (to a given stress); Trx, thioredoxin.*

### Phosphorylation

Phosphorylation is the addition of a phosphate group to side amino acid chains with free hydroxyl group, i.e., serine (S), threonine (T, more frequent), and tyrosine (Y, less frequent), leading to the introduction of a negative charge and decrease in pI value of the protein. In addition to amino acids with a free hydroxyl group, phosphorylation of histidine residues resulting in phosphohistidine was also reported mostly on prokaryotes and lower eukaryotes such as yeast. However, phosphohistidine reveals stability lower than that of amino acids with phosphorylated hydroxyl groups; it, thus, represents a transient intermediate in some reactions.

Phosphorylation represents the most studied PTM at the plant proteomic level. currently, phosphoproteomics studies were published on salt-treated *Arabidopsis thaliana* membrane fraction (Hsu et al., 2009), osmotic stress-treated *A. thaliana* (Xue et al., 2013), salt-treated rice (Chitteti and Peng, 2007), drought-treated rice (Ke et al., 2009), Cd-treated rice (Zhong et al., 2017; Fang et al., 2019), chickpea under dehydration stress (Subba et al., 2013; Barua et al., 2019), wheat embryo and endosperm exposed to salinity (Luo et al., 2019), wheat seedling leaves and grains exposed to osmotic stress (Zhang et al., 2014a,b), wheat flag leaves exposed to dehydration (Luo et al., 2018), wheat embryo and endosperm under salinity (Luo et al., 2019), wheat spikes infected by *Fusarium graminearum* (Ding et al., 2016), einkorn wheat leaves exposed to salinity (Lv et al., 2016), barley root under salinity (Horie et al., 2011), *Brachypodium distachyon* exposed to drought (Yuan et al., 2016), soybean root tips exposed to flooding (Yin and Komatsu, 2015), maize leaves subjected to drought, heat, and their combination (Hu et al., 2015b), maize leaves under osmotic stress (Hu et al., 2015a), maize and drought (Bonhomme et al., 2012), common bean exposed to osmotic stress (Yang et al., 2013), sugar beet exposed to salinity (Yu et al., 2016), okra seedlings exposed to salinity (Yu et al., 2019), *Picea wilsonii* pollen subjected to sucrose deficit (Chen et al., 2012), and others as listed in Table 3.

Protein phosphorylation is catalyzed by protein kinases while protein dephosphorylation is catalyzed by protein phosphatases. Protein phosphorylation rates are quite low and reach only 1–2% of the entire protein abundance, especially when regarding transient protein phosphorylation in signaling pathways. Moreover, there are also differences in phosphorylation rates among different amino acid residues, indicating that some residues are quantitatively more phosphorylated while others, such as tyrosine residues, may be transiently phosphorylated only by up to 0.5%. Although tyrosine-phosphorylated proteins exist in relatively low abundance, which is 4.2% in Arabidopsis, 2.9% in rice, and 1.3% in Medicago, Y- phosphorylation plays an important role in plant responses to a changing environment (Hashiguchi and Komatsu, 2016). Due to the low phosphorylation level of target proteins, a phosphoprotein fraction has to be enriched prior to further analyses, such as using Ti^4+^ IMAC beads. An overview of phosphoprotein enrichment methods is provided by Fíla and Honys (2012) and Silva-Sanchez et al. (2015).

Phosphorylation was most studied in the context of protein signaling. Multiple-step protein phosphorylation cascades, such as the MAPKKK-MAPKK-MAPK kinase cascade, enable initial signal amplification during their transfer in plasmalemma-to-nucleus direction (Yamaguchi-Shinozaki and Shinozaki, 2006). In addition, calcium-dependent protein kinases are also involved in stress signaling and stress response induction. Cieśla et al. (2016) reported that Arabidopsis plants overexpressing barley calcium-dependent kinase HvCPK2a with autophosphorylation sites displayed enhanced drought sensitivity, indicating that HvCPK2a is a negative regulator of the drought stress response.

Phosphoproteomic studies revealed that phosphorylation does not only play an important role in signaling and signal transduction but also in DNA replication and transcription, stress and defense response to pathogens as well as in the regulation of ROS scavenging and cell metabolism. For example, Fang et al. (2019) reported differential phosphorylation of replication-related proteins such as DNA replication/repair enzymes, DEAD-box ATP-dependent RNA helicase 37, and transcription factors (TFs) such as MYB, zinc-finger, and F-box TFs in Cd-tolerant rice line with respect to a sensitive one. Thus, it seems that differential phosphorylation may alleviate adverse Cd impacts on replication and transcription. Luo et al. (2018) found differential phosphorylation of photosynthesis (LHCII, RubisCO activase) and energy metabolism-related (FBA, GAPDH, pyruvate decarboxylase complex components) proteins in wheat flag leaf under drought. Similarly, differential phosphorylation in photosynthesis antenna proteins and proteins involved in RNA degradation was found in salt-treated okra seedlings (Yu et al., 2019). Ding et al. (2016) studied wheat phosphoproteome as affected by *F. graminearum* infection and found alterations in phosphorylation of cinnamoyl-CoA reductase, isochorismate synthase, cytochrome P450, peroxidase 8, and glutathione reductase (GR) involved in lignin and phytoalexin biosynthesis as well as in ROS scavenging. Similarly, Zhang et al. (2014a) identified significant differences at phosphorylation level in proteins involved in signaling and signal transduction, RNA transcription and processing, and stress/defense pathways in two wheat cultivars exposed to osmotic stress due to PEG-6000 treatment. Phosphoproteins involved in ABA signaling include SnRK2 kinases and PP2C phosphatases, and in Ca^2+^ signaling include components of phosphatidylinositol-4,5-diphosphate pathway and CDPK, calmodulin2-2 and calcineurin B-like protein-interacting protein kinases (CIPKs), as well as components of MAPK signaling cascades. Their activities are often regulated by the phosphorylation status of other proteins with regulatory functions such as 14-3-3 proteins (Yu et al., 2016). Phosphorylated TFs include TFs involved in ABA-dependent pathways, such as TaABI5-1, which is phosphorylated by SnRK2 and dephosphorylated by PP6 and binds to ABRE motifs in COR/LEA gene promoters. Other ABA-dependent TFs undergoing phosphorylation are MYB TFs such as MYB1R1 and TaMYB2A. Besides ABA-dependent pathways, zinc finger CCCH domain TFs were found phosphorylated at serine residues under osmotic stress. Yin and Komatsu (2015) identified 14 differentially abundant phosphoproteins in soybean root tips exposed to flooding including nuclear-localized ABA-responsive phosphoproteins such as zinc finger/BTB domain-containing protein 47, glycine-rich protein, and rRNA-processing protein Rrp5. Similarly, Yin et al. (2014) reported a significant effect of ethylene signaling on phosphoproteome in soybean root tips under flooding, resulting in enhanced dephosphorylation of eukaryotic translation initiation factor 4G and enhanced phosphorylation of proteins involved in protein synthesis. Under high salinity, increased phosphorylation level in proteins related to stress and defense response (Cu/Zn-SOD, 2Cys Prx BAS1), carbohydrate metabolism, photosynthesis, and the transport was found in einkorn wheat (Lv et al., 2016). HvPIP2 water transport activity was modulated by phosphorylation under salinity treatment in barley roots (Horie et al., 2011). Similarly, serine phosphomotifs were identified in aquaporins PIP2,1 and PIP2, 4 in salt-treated *A. thaliana* (Hsu et al., 2009).

Barua et al. (2019) studied nuclear phosphoproteome in chickpea subjected to dehydration stress and identified 546 kinases such as those from MAPK kinase cascade, calcium-dependent protein kinases, SRSF protein kinase 1, and others. They also identified differential phosphorylation in E3 SUMO-protein ligase SIZ1-like isoform X1, spliceosome complex, and splicing-related proteins, indicating their altered function under stress. In addition, several regulatory proteins involved in protein degradation and regulation of flowering time and circadian clock revealed dehydration-induced dephosphorylation. A phosphomotif analysis revealed dehydration-induced enrichment in proline-directed Ser phosphorylation. Similarly, Yuan et al. (2016) identified several drought-responsive phosphoproteins involved in signal transduction (MAPK cascade, ABA-responsive SnRK2, Ca^2+^-responsive CDPKs), gene expression, stress and defense, photosynthesis and energy metabolism, and transmembrane transport including six phosphomotifs enriched in drought-treated *B. distachyon*. Hu et al. (2015b) studied maize phosphoproteome response to drought, heat, and their combination and described seven stress-enriched phosphomotifs, out of which two were common to all stresses, two were common for heat, and one was specific to the combined stress. They also found out that differential stress cues can lead to phosphorylation of differential phosphomotifs in the same proteins.

Phosphorylation as a nuclear localization signal (NLS): In dehydrins possessing a stretch of 4–10 serine residues called *S*-segment, phosphorylation of serine residues by SnRK2.10 kinase serves as an NLS (Close, 1997; Brini et al., 2007; Kosová et al., 2011). Enhanced phosphorylation of four dehydrin proteins without change in their relative abundance was found in common bean root tips exposed to PEG-induced osmotic stress (Yang et al., 2013).

Phosphorylation as a means of epigenetic stress memory: Ding et al. (2012) reported the importance of serine 5 (Ser5P) phosphorylation level in RNA polymerase II associated with drought stress memory-related genes in *A. thaliana*. The level of Ser5P in RNA polymerase II remained the same upon recovery as under drought stress in RNA polymerase II associated with drought stress memory-related *COR/LEA* genes *RD29B* and *RAB18*, revealing altered expression pattern under repeated stress when compared to the first stress treatment. Enhanced level of Ser5P in RNA polymerase II associated with drought stress memory-related genes persisting upon stress recovery indicates a stalled state of RNA polymerase II, i.e., its preparedness to transcription initiation upon repeating stress events that persist upon stress recovery when target transcript levels decreased. Differential phosphorylation of histone deacetylases was reported in dehydration-treated maize leaves (Bonhomme et al., 2012).

### Glycosylation

Glycosylation represents the most abundant protein PTM in plants. It represents a highly spatially and temporarily coordinated enzymatic process occurring in the endoplasmic reticulum and Golgi apparatus (proteins belonging to the secretory pathway). Two kinds of protein glycosylation can be distinguished: *N*-glycosylation, where a relatively large oligosaccharide core is bound to an Asn residue, which is then trimmed and modified to a final form, and *O*-glycosylation when mostly just a single monosaccharide is linked to target Ser or Thr residues.

*N*-glycosylation-target amino acids represent asparagine (Asn) residues present in Asn-X-Ser/Thr motif, where X represents any amino acid except proline. In the first step, the basic tetradecyl glycan precursor GlcNAc_2_Man_9_Glc_3_ (Glc for glucose, Man for mannose, and GlcNAc for *N*-acetylglucosamine) is transferred to target asparagine residue by oligosaccharyl transferase complex (OST) in endoplasmic reticulum (ER) and the bound oligosaccharidic core is further trimmed and modified in Golgi apparatus by class I α mannosidases MNS1 and MNS2, which cleave α-1,2-mannosyl residues to generate the substrate for CGL1/GnT1, which catalyzes a GlcNAc addition to remove two additional mannose residues, to add another one GlcNAc, xylose, and fucose residues to form complex *N*-glycan structure (Strasser et al., 2004). Kang et al. (2008) reported a crucial role of *N*-glycoprotein maturation, such as *complex glycan 1* (*cgl1*), for plasma membrane glycoproteins involved in cellulose biosynthesis and cell wall formation in salt-treated *A. thaliana*. Recently, a role of protein *N*-glycosylation in response to salinity stress was studied in *A. Thaliana* wild-type and two mutants, *mns1mns2* and *cgl1*, defective in *N*-glycan maturation leading to the identification of salt-responsive *N*-glycoproteins such as class III peroxidases PRX32 and PRX34, which appeared to be involved in root growth and development under salinity stress (Liu et al., 2021).

*N*-glycoproteins were also studied using a concanavalin A lectin blot technique in cold-treated rice leaf sheaths (Komatsu et al., 2009) and in flooded soybean roots (Mustafa and Komatsu, 2014). In cold-treated rice leaf sheaths, 22 glycoproteins were determined by the lectin blot, out of which 12 revealed significant cold response; cold-responsive *N*-glycoproteins included calreticulin involved in Ca^2+^ signaling and several proteins involved in energy metabolism (mitochondrial F1-ATPase, 6-phosphogluconate dehydrogenase, NADP-dependent malic enzyme, emolase, UDP-glucose pyrophosphorylase) and protein folding (CPN60α, HSP90; Komatsu et al., 2009). In flooded soybean root tips, the accumulation of *N*-glycoproteins related to protein degradation, cell wall, and glycolysis increased, while glycosyl hydrolases, peroxidases, and other proteins related to protein glycosylation pathway, stress and defense response, and protein degradation decreased upon flooding with respect to control plants. In contrast, glycoproteins involved in glycolysis were activated (Mustafa and Komatsu, 2014). However, generally, an overall decrease in glycoprotein accumulation was found in flooded soybean root tips because of a decrease in ER-bound CNX/CRT lectin complex involved in *N*-glycoprotein processing (Wang and Komatsu, 2016b).

Probably the most studied protein *O*-glycosylation is *O*-GlcNAcylation, i.e., reversible addition and removal of single *O*-linked-β-*N*-acetylglucosamine (*O*-GlcNAc) activated as UDP-GlcNAc to target Ser or Thr residues mediated by *O*-GlcNAc transferases (OGTs) including two classes, SPINDLY (SPY) and SECRET AGENT (SEC), and *O*-GlcNAcases (OGAs).

Lectins represent an important protein group interacting with glycoproteins and, thus regulating their biological functions. One of the molecular mechanisms regulating vernalization duration in winter-type Triticeae lies in *O*-GlcNAc modification of Thr17 in a glycine-rich RNA-binding protein TaGRP2, which binds to *VRN1* pre-mRNA first intron region, resulting in *VRN1* transcript repression. During vernalization, the level of *O*-GlcNAc in TaGRP2 increases, leading to enhanced interaction with Jacalin lectin VER2, thus, leading to enhanced TaGRP dissociation from *VRN1* pre-mRNA first intron, resulting in enhanced *VRN1* transcript accumulation. *O*-glycosylation modification, thus, regulates VER2-TaGRP2 protein-protein interaction, resulting in altered interaction of VER2 with *VRN1* first intron region and regulation of the major vernalization gene *VRN1* expression (Xiao et al., 2014). *O*-glycosylation has also been reported for some dehydrins in cold-treated blueberry (Levi et al., 1999) and pistachio (Yakubov et al., 2005), although no specific functions for glycosylated forms of these proteins have been suggested.

### Methylation

Histone methylation in arginine and lysine ε-amino groups *via S*-adenosylmethionine (SAM) as a universal methylating agent represents an epigenetic modification, resulting in either repression or activation of target gene expression. Methylations resulting in target gene repression include H3K9me2 (histone H3 lysine 9 dimethylation), H3K27me3 (histone H3 lysine 27 trimethylation), and H4R3me2 (histone H4 arginine 3 symmetrical dimethylation), while methylations leading to target gene transcriptional activation include H3K4me2 (histone 3 lysine 4 dimethylation) and H3K4me3 (histone H3 lysine 4 trimethylation) modifications. Histone 3 lysine K9 dimethylation (H3K9me2) followed by binding of polycomb-group (PcG) complexes results in repression of a target gene, such as *FLOWERING LOCUS C (FLC)*, a major flowering repressor in *A. thaliana*, following vernalization fulfillment, thus enabling activation of flowering-inducing pathway genes *SOC1* and *FT* (Sung and Amasino, 2005). In contrast, vernalization fulfillment in Triticeae cereals leads to a decrease in H3K27me3, while an increase in H3K4me3 at the 5′end of intron 1 region results in activation of the Triticeae *VRN1* gene (Oliver et al., 2009). Similarly, the H3K4me3 of “trainable genes” revealing enhanced expression under repeated drought stress in comparison to the first drought treatment was also described as a means of drought stress memory in *A. thaliana* (Ding et al., 2012).

### Acylation

#### Short Carboxylic Acids

Protein acylation, i.e., the addition of a relatively short hydrophilic negatively charged acyl group to a positively charged lysine residue, results in significant modifications of target lysine chemical properties. Lysine acylation/deacylation is enzymatically catalyzed by lysine acyltransferases (KATs)/sirtuin-class lysine deacylases (KDACs), respectively, and includes lysine acetylation, propionylation, butyrylation, β-hydroxybutyrylation, malonylation, glutarylation, succinylation, and crotonylation, which significantly modulate cellular metabolism, namely photosynthesis (Hirschey and Zhao, 2015).

Histone acylation of lysine ε-amino group represents an epigenetic modification resulting in the activation of target gene expression. Histone lysine acylation includes acetylation, propionylation, butyrylation, β-hydroxybutyrylation, malonylation, succinylation, and crotonylation employing acylCoA as an acylating agent. Crotonylation could be targeted to all histones, H1, H2A, H2B, H3, and H4. Lysine acetylation in histone H3 results in the activation gene expression such as in the case of the major vernalization gene *VRN1* inducing flowering in Triticeae (Oliver et al., 2009).

Protein succinylation involves targeted succinylation of lysine residues in conserved motifs. Recently, Wang et al. (2021) studied barley succinylome in response to short-term phosphate (Pi) starvation and the following recovery using an immunoblot approach with a specific antibody raised against succinylated lysine. They identified 2,840 succinylation sites across 884 proteins, of which 11 conserved succinylation-related protein motifs were determined. Pi starvation enriched protein succinylation of ribosomal proteins (40S ribosomal protein S6, 60S ribosomal proteins L4-1, L6), proteins involved in glycolysis, protein translation (eukaryotic translation initiation factor 5A-1 EIF5A, elongation factors 1-α and Ts), and RNA degradation, while Pi recovery enriched lysine succinylome in TCA cycle, glycolysis (GAPDH), and oxidative phosphorylation (ATP synthase subunits β and δ of FoF1-ATPase) pathways.

#### Long Carboxylic Acids (Fatty Acids)

Addition of long hydrophobic fatty acid chains, such as palmitic acid (C16:0) or myristic acid (C14:0) (palmitoylation, myristoylation), to membrane proteins provides a hydrophobic “anchor” attaching the protein to hydrophobic lipid bilayer. *N*-myristoylation of lysine residues in target proteins affects protein-membrane interactions (McLaughlin and Aderem, 1995), thus modulating the function of membrane-associated proteins involved in stress signaling, such as SOS3 function (Ishitani et al., 2000) and SnRK1 kinase pathway (Pierre et al., 2007). Moreover, *N*-myristoylation also modulates the function of proteins involved in ubiquitin-dependent protein degradation, namely, F-box proteins and parts of 26S proteasome as well as MYB transcription factors (Boisson et al., 2003; Podell and Gribskov, 2004).

### Peptide Conjugation

#### Protein *S*-Glutathionylation

*S*-glutathione is a three amino acid peptide (γ-glutamyl-cysteinyl-glycine) ubiquitously found in cell cytosols where it acts as a cellular redox state regulator because of reversible GSH ↔ GSSG transitions. Recently, it was found that glutathione conjugation to cysteine residues in cellular proteins acts as a protection shield against ROS, thus preventing protein oxidative modifications (Diaz-Vivancos et al., 2015). Martinez-Seidel et al. (2021) found enhanced levels of enzymes involved in glutathione biosynthesis (hydroxyacylglutathione hydrolase-like) and *S*-glutathione conjugation (five members of glutathione *S*-transferase family) in barley root tips subjected to cold acclimation, indicating a protective role of protein *S*-glutathionylation against oxidative stress associated with most environmental stresses.

#### Protein Ubiquitination

Ubiquitin is a small peptide (8.6 kDa, 76 amino acids) attached to ε-amino group in lysine residues of a target protein by enzyme-regulated series of reactions called ubiquitination. Ubiquitination is a three-step process that includes ubiquitin activation, conjugation, and ligation of ubiquitin to target lysine residues *via* a diglycine motif in ubiquitin C-terminus catalyzed by ubiquitin activase (E1), ubiquitin conjugase (E2), and ubiquitin ligase (E3) enzymes, respectively. Ubiquitin also underlies automodification in each of its seven lysine residues (K6, K11, K27, K29, K33, K48, and K63), out of which K48-linked polyubiquitination is the predominant type involved in protein targeting to proteasome-mediated degradation (Hershko et al., 2000). Ubiquitination as a mark of 26S proteasome-targeted protein degradation plays an important role in plant stress acclimation, since this process is associated with an enhanced rate of both protein biosynthesis and protein degradation (Sharma et al., 2016). Bueso et al. (2014) reported an interaction of RING-type E3 ubiquitin ligase RSL1 with PYL4 and PYR1 ABA receptors resulting in their targeted degradation, thus implying that the E3 ubiquitin ligase affects ABA signaling in *A. thaliana*. In *A. thaliana*, RING finger E3 ubiquitin ligases AtAIRP4 and SDIR1 were reported to positively modulate salt stress response *via* ABA signaling (Yang et al., 2015; Zhang et al., 2015).

#### Protein Sumoylation

SUMO (a small ubiquitin-like modifier; 12 kDa, 100 amino acids) are small proteins structurally similar to ubiquitin which are covalently attached *via* their C-terminal diglycine motif to lysine ε-amino group in the target proteins *via* an enzymatic cascade of SUMO-conjugating enzymes (SCE) analogous to that involved in ubiquitination, i.e., SUMO-activating enzyme (E1, SAE), SUMO-conjugating enzyme (E2, SCE) nad SUMO-ligase (E3). However, sumoylation does not lead to target protein degradation (Benlloch and Lois, 2018). Ghimire et al. (2020) studied alterations in potato (*Solanum tuberosum*) seven *StSUMO* genes, and nine StSCE enzymes in response to salt and PEG treatments at transcript levels by qRT-PCR and found an upregulation in *StSCE1/5/6/7* under both salt and PEG treatments, while *StSUMO1/2/4* were upregulated only under salt stress, whereas *StSUMO2/4* were downregulated under PEG treatment.

### Protein Posttranslational Modifications by Reactive Molecular Species

Environmental stress leads to an enhanced imbalance in key processes of plant energy metabolism, especially photosynthesis and aerobic respiration, resulting in enhanced production of RMS. According to reactive atoms, major types of RMS include reactive oxygen species (ROS), reactive nitrogen species (RNS), reactive carbonyl species (RCS), reactive sulfur species (RSS), reactive halogen species (RXS), and reactive metal species. RMS-mediated PTMs, thus, represent products of spontaneous reactions, which, in case of amino acids in protein active sites, can potentially lead to loss of target protein biological activity. RMS-mediated PTMs have recently been reviewed by Mock and Dietz (2016). ROS, RNS, and RSS in legumes subjected to abiotic stresses were discussed by Matamoros and Becana (2021).

#### Reactive Oxygen Species

Reactive oxygen species (ROS) include superoxide anion radical, hydroxyl radical, hydrogen peroxide, singlet oxygen. Major ROS in plant cells arise from imbalance at acceptor sides of photosynthetic electron transport chain and respiratory electron transport located in inner membranes of chloroplast and mitochondria, respectively. Photosynthetic electron transport chain reveals significant alterations in absorbed light energy leading to electron excitation and the following transport in response to sunlight whose intensity can significantly change within fractions of a second. Excess electrons at the acceptor side of the photosynthetic electron transport chain react with oxygen (the so-called Mehler reaction/Fenton reaction), resulting in superoxide anion formation. ROS also react with a wide array of target amino acids such as sulfur-containing cysteinyl and methionyl residues (Cys, Met), His, Trp, and Tyr, resulting in the formation of peroxide or dioxetane intermediates. Oxidized Tyr and Trp convert to indole derivatives. Histidyl endoperoxides can form His-His or His-Lys crosslinks. Peroxidized Met residues convert to methionine sulfoxide (Mock and Dietz, 2016).

#### Reactive Carbonyls: Protein Glycation

Protein glycation represents a spontaneous non-enzymatic modification of side free amino groups in target amino acids lysine, arginine, proline, and threonine by active carbonyl groups found in some metabolic intermediates (active dicarbonyls such as glyoxal and methylglyoxal, MG) and reducing sugars, such as glucose, through the so-called Maillard reaction. The reaction is spontaneous and can lead to loss of protein biological activity, since target lysine and arginine residues prone to glycation are often found in protein active sites and domains underlying protein biological activity. Moreover, the risk of protein glycation increases under stress because of enhanced levels of active dicarbonyls glyoxal, MG, and 3-deoxyglucosone as major glycating agents. In contrast, sucrose, as a main sugar transport form in plants, is a non-reducing sugar (no active carbonyl group); thus, it does not reveal a glycating activity (Suzuki et al., 2010; Rabbani et al., 2020).

Major glycation adducts represent Nε-(1-deoxy-D-fructose-1-yl) lysine (FL), MG-derived hydroimidazolone (MG-H1; arginine-derived adduct from MG), Nε-carboxymethyl-lysine (CML), glucosepane (formed by degradation of FL residues with a proximate arginine residue), and pentosidine [for more details, see review by Rabbani et al. (2020)].

Detection of carbonylated proteins in 2-DE gels is usually performed using an OxyBlot kit, which lies in the derivatization of carbonylated amino acids by dinitrophenylhydrazine (DNPH) to form dinitrophenylhydrazones, which can be detected by a specific primary antibody against dinitrophenylhydrazones. Enhanced protein carbonylation detected by DNPH immunoblots was found in cadmium-treated pea (Romero-Puertas et al., 2002).

Protein carbonylation belongs to the earliest studied plant protein PTMs. In *A. thaliana*, studies on carbonylated proteins during the vegetative stage (Johansson et al., 2004) and during seed germination (Job et al., 2005) using an Oxyblot kit for carbonylated protein detection and led to the identification of a wide range of carbonylated proteins, such as HSP70, ATP synthase subunits, and RubisCO LSU, and publication of several glycolytic enzymes. Most stress factors (except flooding causing hypoxia) enhance ROS levels, resulting in enhanced protein carbonylation. Pandey and Dubey (2021) studied the combined effect of PEG-induced dehydration and aluminum toxicity on protein carbonylation in rice seedlings belonging to two contrasting cultivars, Malvyia-36 (sensitive) and Vandana (tolerant), and found differential carbonylation in several photosynthesis-related proteins such as RubisCO LSU, PS I reaction center subunit II and IV proteins, and OEE3 protein, as well as in calmodulin involved in Ca^2+^ signaling.

#### Protein Nitrosylation by Reactive Nitrogen Species

Major reactive nitrogen species (RNS) include nitric oxide (NO), nitrogen dioxide (NOO.), and peroxynitrite (ONOO^–^), which are generated by the reaction of NO with superoxide radicals (O_2_^–^) and oxygen, respectively. Nitric oxide (NO) is synthesized by cytosolic nitrate reductase as a side product and reacts with thiol groups in Cys residues, resulting in the formation of *S*-nitrosothiols, while peroxynitrite and nitrogen dioxide are major NO donors for Tyr nitration. The major buffer for NO in plant cells is glutathione (GSH), resulting in the formation of *S*-nitrosoglutathione (GSNO). Recently, the molecular mechanisms involved in protein *S*-nitrosylation and current knowledge of the biological roles of *S*-nitrosoproteins in plants were reviewed by Feng et al. (2019).

Abat and Deswal (2009) studied NO-induced *S*-nitrosoproteome in *Brassica juncea* treated with various environmental stresses such as cold, drought, heat, and salinity, and reported *S*-nitrosylation (SNO)-related inactivation of RubisCO carboxylase activity under cold stress. RubisCO depletion led to the identification of *S*-nitrosylation in further proteins such as thioredoxin, SOD, GAPDH, FBA, and sedoheptulose bisphosphatase, indicating an important role of *S*-nitrosylation in the regulation of Calvin cycle enzymes in cold-treated *B. juncea* seedlings (Sehrawat et al., 2013). In addition, further study on *S*-nitrosoproteins in cold-treated *B. juncea* seedlings (Sehrawat and Deswal, 2014) identified novel targets such as storage proteins (12S globulin cruciferin), stress- and defense-related proteins (lactoylglutathione lyase, germin-like protein), and redox metabolism-related proteins (DHAR, Cu/Zn-SOD). Camejo et al. (2013) reported enhanced *S*-nitrosylation of several mitochondrial proteins in salt-treated pea, namely ATP synthase β subunit, Hsp 90, and peroxiredoxin IIF (PRXIIF), leading to a decrease in PRXIIF enzymatic activity.

Tanou et al. (2009, 2012) studied the effects of hydrogen peroxide (H_2_O_2_) and nitric oxide ^(^NO) as an ROS and an RNS, respectively, on citrus (*Citrus aurantium*) plants exposed to salinity and identified 40 carbonylated and 49 *S*-nitrosylated proteins, indicating an overlap between H_2_O_2_ and NO-induced signaling pathways. Ziogas et al. (2013) studied the impacts of six abiotic stresses namely, cold, heat, drought, salinity, continuous light, and darkness on citrus plants with respect to their nitrosoproteome and RNS levels and found differential impacts of the different stresses on RNS and related enzymes activities. Cold led to enhanced *S*-nitrosoglutathione reductase (GSNOR) and nitrate reductase (NR) gene expression and enzymatic activity. Peroxynitrite scavenging activity was elicited by continuous light, darkness, or drought but it was suppressed by salinity. Nitration activity was enhanced by salinity and suppressed by continuous light or darkness. Protein *S*-nitrosylation levels were enhanced by heat, cold, and drought but were suppressed by darkness and salinity.

The impacts of heat stress on tyrosine nitration (Y-nitration) were studied in sunflower by Chaki et al. (2011, 2013) who found that tyrosine nitration leads to the inhibition of ferredoxin-NADP reductase and β-carbonic anhydrase (β-CA) enzymatic activities, respectively, implicating adverse impacts of heat on photosynthesis *via* enhanced RNS.

#### Protein Modification by Reactive Sulfur Species

Major reactive sulfur species (RSS) include thiyl radicals, sulfenic acid, and disulfide monooxide.

Protein persulfidation, also known as protein sulfhydration, represents a modification of thiol groups (-SH) in target cysteine residues to -SSH by H_2_S, which is produced from cysteine in stomatal guard cells by the L-cysteine desulfhydrase 1 (DES1) enzyme, which is involved in ABA-dependent signaling pathway regulating stomatal closure. Enhanced levels of persulfidated proteins were found as being associated with autophagy in response to severe environmental stresses such as drought and nutrient starvation (e.g., nitrogen starvation). Protein persulfidation can be detected by tag-switch label-free LC-MS/MS approach. Persulfidation was reported for H_2_S-dependent PTM of SnRK2.6 kinase in ABA-dependent signaling pathway and DES1 itself. Persulfidation was reported for several proteins involved in ABA-dependent signaling pathways such as ABA receptors PYR/PYL and their downstream elements such as SnRK2.1 protein kinase and PP2C-7 protein phosphatase as well as proteins involved in jasmonic acid (JA) and brassinosteroid (BR) signaling, BRI1 or BSK1. Several proteins involved in signaling related to autophagy, such as SNF1-related protein kinase catalytic subunits alpha KIN10 and KIN11 and receptor-like kinases FERONIA and THESEUS1, were found persulfidated under N starvation in *A. thaliana* (Jurado-Flores et al., 2021). Protein persulfidation plays a role in the processes of autophagy, ubiquitin-dependent protein degradation, and ABA-dependent signaling.

## Non-Covalent Protein Modifications

In addition to covalent PTMs, non-covalent protein modifications such as protein/enzyme cofactors and/or substrates resulting in different protein/enzyme conformations represent a further level leading to novel functional proteoforms. Proteins/enzymes with multiple cofactors/substrates, probably the most studied plant enzyme with multiple substrates and multiple catalytic functions, is RubisCO, i.e., ribulose-1,5-bisphosphate carboxylase/oxygenase, which can bind either CO_2_ or O_2_ as competing substrate and, thus, catalyze either carboxylation or oxygenation of the substrate ribulose-1,5-bisphosphate. RubisCO needs RubisCO activase (RCA) to activate the RubisCO active site for CO_2_ binding.

Enzymes can modify their affinity to a substrate based on ambient substrate concentration. In oligomeric enzymes with multiple catalytic sites, allosteric effect occurs, i.e., transition of the catalytic site from a low-affinity state to a high-affinity state is transmitted from one subunit to the other. Allosteric regulation, thus, means the spatial effect of the subunit on the others in oligomeric enzymes. This type of regulation of the affinity of the binding site is not possible in monomeric enzymes. However, it was also found that in monomeric enzymes, such as glucokinase, increasing glucose concentration as a substrate leads to an increased shift of the enzyme from low-affinity to high-affinity state analogously to the allosteric regulation in oligomeric enzymes (Whittington et al., 2015). As substrate concentration increases, the probability of encountering other substrate molecules after turnover also increases, thus preventing the enzyme from relaxing back to the low-affinity state. This time-dependent shift in the ratio of low-affinity to high-affinity enzyme proteoforms with respect to increased substrate concentration is called allokairy (Hilser et al., 2015). Mnemonical enzymes are monomeric enzymes that can retain high-affinity conformation after product release when substrate levels increase in an ambient environment (Ricard et al., 1974). The shifts between enzyme low-affinity and high-affinity states, such as allokairy in monomeric human glucokinase, play an important role in disease development (Whittington et al., 2015). However, to the best of our knowledge, no analogous research has been published for plant enzymes.

## Conclusion

Proteins are directly involved in plant responses to environmental cues. Mechanisms of posttranscriptional and posttranslational modifications of protein primary sequence and encoded amino acid residues, respectively, resulting in multiple functional proteins (proteoforms) derived from a single gene. Mechanisms of posttranscriptional and posttranslational modifications increase phenotypic variability with respect to genetic information encoded by the genome. Duplications of local genomic regions, as well as whole genome duplications, and the presence of multiple genomes in polyploid species lead to paralogous and orthologous genes, respectively, which can undergo functional diversifications represented by differential expression patterns. From a biochemical point of view, the twenty gene-encoded amino acids represent a very diverse set of chemical species such as non-polar (hydrophobic) amino acids with aliphatic (alanine, leucine, isoleucine, valine) or aromatic (phenylalanine) side chains as well as both acidic (aspartic and glutamic acid) and basic (lysine, arginine, asparagine, glutamine) groups. The coded amino acids can, thus, undergo several modifications resulting in altered properties. Some PTMs represent a result of a series of spatially and temporally coordinated enzymatic reactions (phosphorylation, *N*-glycosylation, succinylation, crotonylation, ubiquitination, sumoylation), while others occur spontaneously (PTMs arising from reactions with RMS such as oxidation, glycation, and *S*-nitrosylation). The level of spontaneous PTMs rises under environmental stress because of imbalances in cellular metabolism, leading to enhanced formation of reactive molecular species (RMS). RMS-mediated PTMs can result in loss of target protein biological activity, but they also serve as a signal-inducing stress response (Chaki et al., 2011, 2013; Camejo et al., 2013; Mock and Dietz, 2016).

It has to be summarized that PTMs represent a modification of only a relatively small fraction of the given protein; moreover, the modification is often reversible and/or transient, and the resulting PTM, thus, acts as a transient signal of the given stress cue, which is the case of phosphorylation and RMS-induced PTMs. Several proteins can undergo multiple PTMs under the given stress treatment, e.g., calreticulin was reported to undergo both phosphorylation and *N*-glycosylation in cold-treated rice leaf sheaths (Komatsu et al., 2009). Moreover, the same protein can undergo the same kind of PTM in different target motifs under different stress treatments; for example, differential phosphomotifs were identified in the same phosphoproteins in maize phosphoproteome under drought, heat, and their combination (Hu et al., 2015b). All these factors determine the protein’s biological activity.

The major roles of differential protein isoforms and PTMs in plant cells subjected to stress could be summarized as follows (Table 4):

**TABLE 4:** Overview on major protein biological functions affected by different protein isoforms or PTMs in response to environmental stress conditions.

Biological process	Protein isoform/PTM	References
Transcriptome reprogramming under stress	H2AZ histone isoform → altered nucleosomes and DNA wrapping → altered chromatin state resulting in altered transcription	Kumar and Wigge, 2010
Epigenetic stress memory (long-term cold: vernalization; long-term drought)	Epigenetic marks on chromatin proteins: PTMs of histones (methylation, acetylation) and transcription related proteins (RNA polymerase II phosphorylation)	Sung and Amasino, 2005; Oliver et al., 2009; Ding et al., 2012
Stress signaling	Protein signaling cascades (phosphorylation—enzymatically regulated stress signaling; RMS-induced PTMs—spontaneous stress signals)	Yamaguchi-Shinozaki and Shinozaki, 2006; Mock and Dietz, 2016
Differential enzyme activity and kinetics: fine tuning of stress response	*S*-nitrosylation-induced RubisCO inactivation E3 RING-type ubiquitin ligase PARKIN activity: ↑ persulfidation, ↓ *S*-nitrosylation Differential RCA isoforms, differential ROS scavenging enzymes isoforms (Prx, Trx): fine coordination of photosynthetic and mitochondrial electrontransport chains, carbon assimilation	Abat and Deswal, 2009; Vandiver et al., 2013; Wang et al., 2020
Differential protein biological function determined by cellular localization	Cytoplasmic vs. nuclear isoforms of glycolytic enzymes: GAPDH—glycolytic enzyme (cytoplasmic, plastid isoforms), transcriptional repressor (nuclear); eIF5A—translation initiation (cytoplasmic), cell cycle regulation (nuclear isoforms)	Lee et al., 2002; Thompson et al., 2004
Differential protein-protein (protein-nucleic acid) interactions	*A. thaliana* HSC70 protein isoforms: HSC70-1 and HSC70-3 constitutive × HSC70-2—pathogen-inducible, interaction with SGT1 involved in R-gene mediated resistance *O*-glycosylation: *O*-GlcNAc modification at Thr17 in TaGRP2 glycine-rich RNA binding protein enhances its interaction with protein resulting in TaGRP2 dissociation from *VRN1* pre-mRNA first intron →*VRN1* mature transcript accumulation	Noël et al., 2007; Xiao et al., 2014

*GAPDH, glyceraldehyde-3-phosphate dehydrogenase; Prx, peroxiredoxin; RCA, RubisCO activase; RMS, reactive molecular species; Trx, thioredoxin.*

Signaling and signal transduction: Stress-induced signal is transduced and amplified *via* reversible phosphorylation in protein kinase cascade. The reversibility of phosphorylation enables efficient regulation of signal spread and switching off of it when target cellular mechanisms are activated (Yamaguchi-Shinozaki and Shinozaki, 2006).

Transcriptome/proteome reprogramming under stress: Expression of differential histone isoforms and PTMs underlying modular reprogramming of plant transcriptome under stress enables expression of whole arrays of newly induced transcripts (Kumar and Wigge, 2010; Janská et al., 2011); ribosomal isoforms underlying proteome reprogramming under stress (Martinez-Seidel et al., 2021).

PTMs of histones and transcription-associated proteins determine the preparedness of stress memory-related genes to prompt transcription under repeated stress events resulting in enhanced stress tolerance. Stress memory mechanisms include both target gene repressions, such as FLC repression in *A. thaliana* by H3K9me2 and H3K27me3 followed by polycomb group proteins binding (Sung and Amasino, 2005), and target genes activation such as H3K4me3 and H3 acetylation in association with *VRN1* gene activation in Triticeae following vernalization fulfillment (Oliver et al., 2009).

Differential isoforms and PTMs underlie differential enzymatic kinetics/activity and protein-protein interactions under stress treatments. These modifications are crucial for the final protein biological function. For example, the level of *O*-GlcNAc modification at Thr17 in TaGRP2 glycine-rich RNA binding protein determines its interaction with VER2 Jacalin lectin protein, resulting in TaGRP2 dissociation from *VRN1* pre-mRNA first intron and induction of *VRN1* transcript accumulation (Xiao et al., 2014). Phosphorylation of penultimate Thr in plasma membrane H^+^-ATPase leads to binding to phosphorylated C-terminus in 14-3-3 protein involved in the regulation of stomatal aperture in guard cells of *A. thaliana* rosette leaves (Hayashi et al., 2011). In salinity signaling, a physical interaction of myristoylated Ca^2+^-binding protein SOS3 with a Ser/Thr protein kinase SOS2 leads to SOS3-SOS2 complex formation and SOS2-mediated phosphorylation of SOS1 plasma membrane-localized Na^+^/H^+^ antiporter, resulting in its activation (Sanchez-Barrena et al., 2007). In addition, it was found that SOS2 activity is modulated by the phosphorylation status of 14-3-3 protein. Phosphorylated 14-3-3 protein inhibits SOS2 kinase activity, while 14-3-3 dephosphorylation upon salt stress leads to SOS2 activation (Yu et al., 2016).

Differential peroxiredoxin (Prx) and thioredoxin (Trx) isoforms finely tune redox reactions at the end of electrontransport chains in inner membranes of chloroplast and mitochondria. RubisCO activase (RCA) isoforms were found to finely tune RubisCO activity in heat-treated Rhododendron (Wang et al., 2020).

Besides modulation of enzyme activity, protein isoforms and PTMs are also involved in the modulation of structural protein properties such as chaperone Hsc70 isoforms determining other protein conformations and interactions (Noël et al., 2007), and actin isoforms underlying altered thickness of actin filaments (Kijima et al., 2018); altered patterns of *N*-glycosylation in cell wall proteins were found in salt-treated *A. thaliana* (Liu et al., 2021).

**FIGURE 2:**
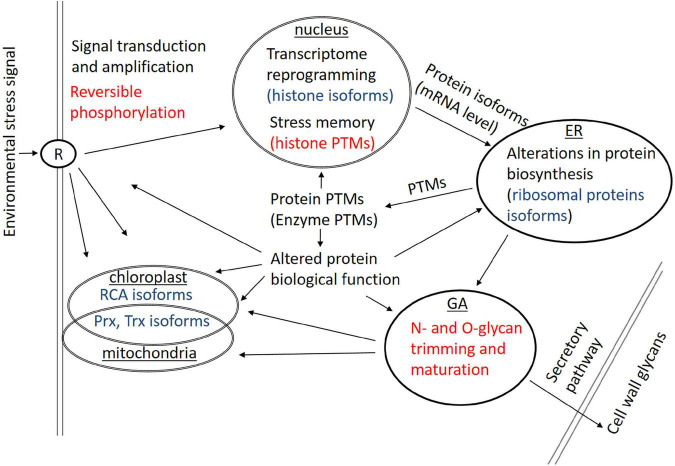
Some examples of cellular processes affected by protein isoforms (blue) and protein posttranslational modifications (PTMs) (red) involved in plant responses to environmental stresses. ER, endoplasmic reticulum; GA, Golgi apparatus; R, receptor protein(s); RCA, RubisCO activase.

It can be concluded that protein isoforms and PTMs are involved in a broad scale of biological processes ranging from cell signaling, transcriptome reprogramming, protein expression, and protein/enzyme function/activity to stress memory mechanisms. Proteoforms represent most probably a universal means of how plants can modulate their biological processes in response to environmental cues; however, the current state of knowledge is far from being complete. Some examples of cellular processes affected by protein isoforms and PTMs in plant responses to environmental stresses are given in Figure 2.

Mechanisms of protein posttranscriptional and posttranslational modifications, thus, represent an efficient means of enhancing biological variability in response to variability in an ambient environment. It is, thus, becoming evident that just the sole identification of protein primary sequence encoded by a given gene does not fully define protein final structure and biological activity. In plants, the study of proteoforms is in its beginnings; however, from comparisons of the diversity of some plant proteins, such as Arabidopsis actin isoforms, with their human homologs, i.e., non-muscle actins, it is evident that plant isoforms reveal greater sequence variability than human isoforms that may reflect the greater diversity of environmental factors that plants, as sessile organisms, have to face during their life cycle (Kijima et al., 2018). When interpreting proteomic results, we have to consider differential proteoforms that can be derived from the identified protein and can reveal differential biological functions. The study of proteoforms, thus, indicates that proteome represents a specific level of an organism that cannot be derived from the simple transformation of the transcriptome. The study of proteoforms also represents a key to understanding the versatility of plant responses to environmental cues such as abiotic and biotic stresses.

## Author Contributions

KK outlined the idea and prepared the draft manuscript text. PV prepared the figures and tables. PV, MK, IP, and JR actively searched for relevant literature, suggested comments on the manuscript draft, and read and approved the final version of the manuscript. All authors contributed to the article and approved the submitted version.

## Conflict of Interest

The authors declare that the research was conducted in the absence of any commercial or financial relationships that could be construed as a potential conflict of interest.

## Publisher’s Note

All claims expressed in this article are solely those of the authors and do not necessarily represent those of their affiliated organizations, or those of the publisher, the editors and the reviewers. Any product that may be evaluated in this article, or claim that may be made by its manufacturer, is not guaranteed or endorsed by the publisher.

## Footnotes

1
http://www.psb.ugent.be/PlantPTMViewer
